# Pyroptosis is involved in ovulation of zebrafish

**DOI:** 10.1038/s41421-021-00263-4

**Published:** 2021-06-01

**Authors:** Zhiquan Liu, Caiyan Niu, Jianzhen Li

**Affiliations:** College of Life Sciences, Northwest Normal University, Lanzhou, Gansu 730070 China

**Keywords:** Cell death, Developmental biology

Dear Editor,

Ovulation involves the release of mature oocytes from the surrounding follicles, which has been characterized as an inflammatory response due to the involvement of immune mediators, such as cytokines and chemokines^1^. Degradation of follicular cells to create a rupture site prior to ovulation is potentially mediated by the programmed cell death, which has been reported in different species^2,3^. Pyroptosis is a novel type of programmed cell death^4^, which is a general innate immune effector process in vertebrates that has dual outcomes. Pyroptosis can protect multicellular organisms from microbial infection and endogenous dangers, but can also result in pathological inflammation if overactivated^5^. Comparing with apoptosis, which is non-inflammatory programmed cell death and executed mainly by Caspase-3, pyroptosis is a pro-inflammatory programmed cell death, which critically depends on the formation of plasma membrane pores by members of the gasdermin protein family, often caused by inflammatory caspase activation. It is unknown whether pyroptosis participates in biological processes other than innate immune defense. In this study, we used zebrafish as a model to demonstrate that pyroptosis is also involved in ovulation.

Similar to other animals, ovulation in zebrafish can be induced by intraperitoneal injection of human chorionic gonadotropin (hCG, from Sigma-Aldrich) (Fig. 1a and Supplementary Fig. S1a). The ovulated oocytes obtained by this ovulation induction system could be fertilized normally (Supplementary Fig. S1b). Thus, the artificially induced ovulation by hCG in zebrafish supplies a convenient model to study ovulation. Propidium iodide (Supplementary Fig. S2) and SYTOX green nucleic acid (Fig. 1b) were used to stain the dead cells in preovulatory follicles during hCG-induced ovulation. Dead cells increased at 2 h (~50%) and 3 h (~80%) after the hCG (20 IU/fish) injection, which was confirmed by quantitative analysis (Fig. 1c). To further assess the type and quantity of the dead cells during ovulation, follicle sections were stained by a terminal deoxynucleotidyl transferase (TdT) dUTP Nick-End Labeling (TUNEL) assay. The signal of TUNEL was significantly increased in the follicular cell layer during ovulation at 2 h after the hCG injection (Fig. 1d, e). These staining results showed that the cell death of follicular cells was increased during the zebrafish ovulation.

**Fig. 1:**
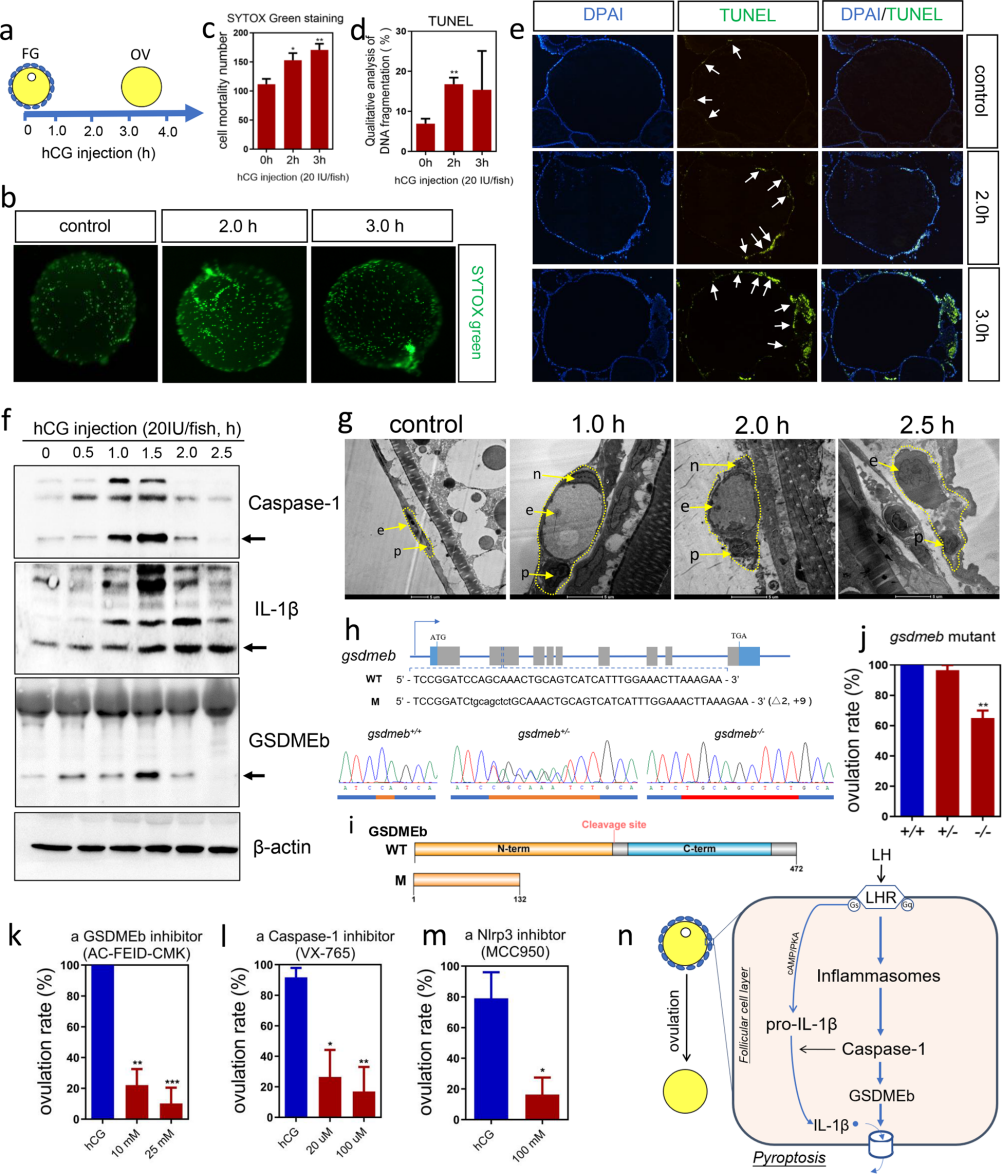
Pyroptosis is involved in ovulation of zebrafish. **a** Ovulation artificially induced by injection of hCG (20 IU/fish) in zebrafish. **b** SYTOX green staining of follicles during ovulation. **c** Statistical analysis of the dead cell number after SYTOX staining. Data are presented as mean ± SEM (*P < 0.05, **P < 0.01, ***P < 0.001 vs 0 h, one-way ANOVA). **d** Statistical analysis of the TUNEL assay. Data are presented as mean ± SEM (**P < 0.01, vs 0 h, one-way ANOVA). **e** TUNEL assay showed the dead cells in follicular cells during ovulation. Signals are indicated by the white arrow. **f** The protein expression changes of Caspase-1, IL-1β, GSDMEb, and β-actin during ovulation detected by western blot. **g** Morphological changes of follicular cells during ovulation of zebrafish viewed by a transmission electron microscope (TEM). n, nucleus of endothelial cell; e, erythrocyte; p, pericyte. Scale bar: 5 μm. **h** Targeted disruption of the zebrafish *gsdmeb* gene by CRISPR/Cas9 system. The above panel shows the location of the gRNA binding site on the *gsdmeb* gene of zebrafish. WT, wild-type; M, homozygous mutant line of F2 generation zebrafish. The below panel shows the sequencing results of *gsdmeb* from wild-type (*gsdmeb*^*+/+*^), heterozygous (*gsdmeb*^*+/−*^), and homozygous (*gsdmeb*^*−/*−^) zebrafish. **i** Schematic protein structure of GSDMEb from wild-type and mutant zebrafish based on sequencing results. **j** The ovulation rate in wild-type, heterozygous and homozygous *gsdmeb* mutant female zebrafish. Data are presented as mean ± SEM (**P < 0.01 vs wild-type, one-way ANOVA). **k**–**m** The effects on hCG-induced ovulation by administration of a Caspase-1-specific inhibitor VX-765 (**k**), a GSDMEb-derived peptide inhibitor Ac-FEID-CMK (**l**), or an Nlrp3-specific inhibitor MCC950 (**m**). Data are presented as mean ± SEM (*P < 0.05, **P < 0.01, ***P < 0.001 vs hCG treatment group, one-way ANOVA for panels **k** and **l**, *t* test for panel **m**). **n** Model of pyroptosis involvement in zebrafish ovulation.

Activation of the NLRP1/3 (NOD-, LRR-, and pyrin domain-containing protein 1/3) inflammasome leads to Caspase-1-dependent release of the pro-inflammatory cytokines IL-1β, and Gasdermin D-mediated pyroptotic cell death^6,7^. During zebrafish ovulation, we found that *nlrp1* and *nlrp3* mRNAs were significantly increased in the preovulatory follicles during hCG-induced ovulation (Supplementary Fig. S3). This is consistent with a mouse study showing that *nlrp3* mRNA was increased in the ovary during ovulation^8^. The activation of Caspase-1 leads to the cleavage of p45 pro-Caspase-1 into the mature fragments p10 and p20^5^. Western blot analysis showed that the active form of p20 Caspase-1 was elevated in the preovulatory follicles during hCG-induced ovulation (Fig. 1f). Activated Caspase-1 can further cleave IL-1β into the active form^5^. Real-time PCR showed that the *il-1β* mRNA was significantly upregulated in the follicular cells during ovulation (Supplementary Fig. S4). This regulation by the LH signal was dependent on the cAMP/PKA pathway (Supplementary Fig. S4e). The active form of IL-1β was also found to be gradually increased during hCG-induced ovulation (Fig. 1f). Two GSDM members in zebrafish, including GSDMEa and GSDMEb, had pore-forming effects in their N-terminal domains^9^. GSDMEa could be cleaved by Caspase-3^9^, but GSDMEb was assumed to be cleaved by Caspase-1^10^. GSDMEb, but not GSDMEa, was activated in the preovulatory follicles during hCG-induced ovulation of zebrafish (Fig. 1f and Supplementary Fig. S5). These results clearly indicated that the expression of most core factors of pyroptosis, including Nlrp1/3, Caspase-1, IL-1β, and GSDMEb, were activated during hCG-induced ovulation in zebrafish. A noncanonical pathway of pyroptosis mediated by a functional analog of Caspase-1 called Caspase-b (Caspy2) has been reported in zebrafish^11^. We detected the active form (p20) of Caspy2 in the postovulatory ovaries but not in the preovulatory follicles (Supplementary Fig. S6). This suggested that this noncanonical pathway of pyroptosis is not involved in zebrafish ovulation.

To exclude a possibility that the commercial hCG reagent was contaminated with infectious pathogens, we used another ovulation-inducing factor called insulin-like growth factor 3 (Igf3)^12^. Similar to the effect of hCG, both Caspase-1 and IL-1β were activated in the preovulatory follicles by administration of recombinant zebrafish Igf3 protein (Supplementary Fig. S7). More importantly, we further demonstrated the involvement of pyroptosis during the natural ovulatory cycle. Using SYTOX green nucleic acid staining, dead cells increased in preovulatory follicles during the natural ovulation cycle (Supplementary Fig. S8). Similar to the results from hCG-induced oocyte maturation, we also found that Nlrp1, Caspase-1, IL-1β, GSDMEb but not GSDMEa was activated during the natural ovulatory cycle of zebrafish (Supplementary Fig. S8).

To further identify which cell types underwent pyroptosis in the preovulatory follicles during ovulation, we used transmission electron microscopy for examination. After hCG administration (20 IU/fish), the endothelial cells of the capillary located in the thecal cell layer were swollen at 1.5 h. The nuclei of the endothelial cells were condensed. The cell membrane began to rupture at 2 h. At 2.5 h, the cell membrane lost its integrity and ruptured. The contents were released with only erythrocytes and pericytes left (Fig. 1g). These results indicated that the vascular endothelial cells in the thecal cell layer died. The morphological changes, including swelling and the rupture of cells, are consistent with the characteristics of pyroptosis^4^.

To study the role of pyroptosis in ovulation of zebrafish, GSDMEb gene knockout zebrafish line was established using CRISPR/Cas9 system (Fig. 1h, i). The ovulation rate was significantly decreased in homozygous mutant lines, by comparing with its corresponding control groups including wild-type and heterozygous mutants with the same developmental stage, body length, and weight (Fig. 1j). This result suggests that GSDMEb is involved in ovulation of zebrafish. We then tested the effect of a GSDMEb-derived peptide inhibitor (Ac-FEID-CMK) on ovulation of zebrafish. The cleavage of GSDMEb during zebrafish ovulation could be totally blocked by administration of Ac-FEID-CMK (Supplementary Fig. S9). Comparable to the effects on ovulation in GSDMEb mutant zebrafish, the Ac-FEID-CMK treatment significantly blocked hCG-induced zebrafish ovulation (Fig. 1k). Furthermore, a specific Caspase-1 inhibitor (VX-765) and a specific Nlrp3 inhibitor (MCC950) were further used to investigate the role of pyroptosis in ovulation. The hCG-induced ovulation was largely attenuated by in vivo administration of VX-765 or MCC950, respectively (Fig. 1l, m). These results strongly suggest that pyroptosis plays an indispensable role in zebrafish ovulation.

Pyroptosis promotes the secretion of IL-1β, the importance of which in ovulation has already been demonstrated in mammals^13,14^, e.g., in rat IL-1β could induce ovulation and the ovulation could be blocked by inhibition of IL-1β signaling pathway. In zebrafish, no obvious effects on ovulation were found using human recombinant IL-1β protein, which might be due to the low conservation of IL-1β activity from zebrafish to human. Therefore, we prepared a recombinant zebrafish IL-1β protein using a bacterial system (Supplementary Fig. S10). Interestingly, inhibitory but not stimulatory effect on hCG-induced oocyte maturation and ovulation was found by administration IL-1β protein (125 ng/fish and 3 ng/fish) in vivo (Supplementary Fig. S10). This inhibitory effect on oocyte maturation was further confirmed by in vitro treatment assays (Supplementary Fig. S10). Thus, we proposed that the proper levels of IL-1β level might be essential for ovulation of zebrafish. Secretion of IL-1β at a low level stimulated by LH signaling might be needed for ovulation, but a high level of IL-1β stimulated by overactivation of inflammatory reaction is inhibitory for ovulation in zebrafish. A recent study in zebrafish also showed the proper levels of IL-1β are critical in tissue regeneration, that the transient IL-1β level is needed for tissues to regenerate, but the prolonged IL-1β expression stimulated by a more severe inflammatory response can block the process^15^.

We also assessed the involvement of two other types of programmed cell death, including apoptosis and autophagy in zebrafish ovulation. The expression of several apoptosis-related and autophagy-related genes was evaluated. Our data did not support the involvement of apoptosis and autophagy in zebrafish ovulation (Supplementary Figs. S11 and S12).

The collective evidence provided herein indicates that pyroptosis is involved in zebrafish ovulation. A model of pyroptosis participation in ovulation is thus proposed (Fig. 1n). Pyroptosis appears to contribute to the weakening of the ovarian follicle wall and facilitates its localized degradation. The inflammatory reaction stimulated by pyroptosis helps to repair ovary damage after ovulation. Although the involvement of pyroptosis in ovulation of other species, including mammals, is unknown, the up-regulation of some core factors of pyroptosis such as NLRP3, Caspase-1, and IL-1β, during ovulation^8,14^, and the importance of Caspase-1 and IL-1β in ovulation has been reported in mammals^13^. These studies suggest that the pyroptosis-related pathway might play a conserved role in the ovulation of species from fish to mammals. In conclusion, we demonstrated that pyroptosis is involved in ovulation of zebrafish. This is in addition to the conventional view that pyroptosis is only associated with differential pathophysiological outcomes in infectious and chronic inflammatory diseases.

## Supplementary information

SUPPLEMENTAL MATERIAL
