# Supplementary material for: Pyroptosis is involved in ovulation of zebrafish
Source: Cell Discov. 2021 Jun 1;7:40. doi: 10.1038/s41421-021-00263-4 (PMC8169692; doi:10.1038/s41421-021-00263-4)
Supplement: Supplementary file 1 — SUPPLEMENTAL MATERIAL [file 41421_2021_263_MOESM1_ESM.docx]

**Supplementary information**

**Materials and Methods**

**Animals**

All the AB strain zebrafish used in this study were purchased from China Zebrafish Resource Center (Wuhan, China). Luteinizing hormone beta subunit mutant (*lhb^-/-^*) zebrafish was established in our previous study (*1*). Fish were maintained under a 15:9 h (L:D) photoperiod in circulating freshwater aquaria at 26°C-28°C. Fish were fed twice daily with newly hatched brine shrimp (Hongjie Brand, Beijing, China). Fish experiments were conducted in accordance to the regulations of the Animal Experimentation Ethics Committee of Northwest Normal University.

**Chemicals and antibodies**

hCG, IBMX, Forskolin, 25% glutaraldehyde were obtained from Sigma-Aldrich (St. Louis, MO, USA), Medium-199 (M-199) and Leibovitz (L-15) culture media, Trypsin, Fetal bovine serum (FBS), fungizone and gentamicin were obtained from Gibco (Amarillo, TX, USA), TUNEL (In Situ Cell Death Detection Kit) was purchased from Roche (Basel, Switzerland). Propidium iodide (PI) and SYTOX Green nucleic acid dye from Invitrogen (Carlsbad, CA, USA). The antibodies for β-actin, Caspase-1 and Caspase-9, HRP-labeled goat anti-mouse IgG, goat anti-rabbit IgG were all purchased from Cell Signaling Technology (Danvers, MA, USA). The antibodies of zebrafish Caspase-1b, GSDMEa and GSDMEb were the gifts from Prof. Dahai Yang at East China University of Science and Technology. Beclin1, Bax and Bcl-2 antibodies were purchased from Proteintech (Wuhan, China). LC-3A antibody was purchased from GeneTex (Irvine, CA, USA). IL-1β antibody was purchased from Abcam (Cambridge, MA, USA).

**PI and** **SYTOX green nucleic acid staining**

The fluorescent dye PI (Propidium Iodide) and SYTOX Green nucleic acid dye are nuclear staining reagents that can stain DNA and is commonly used for dead cell detection. After hCG injection (20 IU/fish, 0 h, 2 h, 3 h), follicles at different time points of ovulation were separated gently and placed in 24-well culture plates (30 follicles/well), stained with PI (10ug/ml) or SYTOX (50μM) for 20 min at room temperature, followed by washing 5 times with PBS for 5 min each time, then photographed by a fluorescence microscope (Olympus, Tokyo, Japan). The excitation and emission wavelengths of the PI-DNA complex are 535 nm and 615 nm respectively. The excitation and emission wavelengths of the SYTOX green are 488 nm and 523 nm respectively. For SYTOX green nucleic acid staining, 20 follicles per group was used to determine the number of died follicular cells in each follicle using ImageJ software.

**TUNEL staining**

Tissue sections were labeled using TUNEL (Tdt-mediated dUTP Nick End Labelling) to detect the breakage of nuclear DNA during cell death. Briefly, the adult zebrafish ovary was collected at different time points after hCG injection (20 IU/fish, 0h, 2h, 3h), embedded in optimal cutting temperature compound (OCT, sakura, Japan) agent, frozen at -25°C, sections were cut in 7 μm thickness on a freezing microtome (Leica CM1520, Germany). The sections on the slides (Thermo scientific, USA) were air dried for 30 min, then fixed with 4% paraformaldehyde in PBS (pH7.4) for 20 min, and washed 3 times with PBS for 10 min. It was then treated with 0.1% Triton X-100 for 5 min and washed twice with PBS for 10 min, use a filter paper to absorb excess water, add 50μl of TUNEL reaction solution (prepared before use), cover with a suitable size of sealing film, and incubate for 1h protected from light. The cell nucleus were visualized with 4’,6-diamidino-2-phenylindole (DAPI) (Solarbio, Beijing, China) counterstaining, incubated in the dark for 30 minutes and photographed by a fluorescence microscope (Leica, Wetzlar, Germany). For control experiments, sections were incubated with PBS. In each group, 15 preovulatory follicles were randomly chosen from different sections to determine the number of died follicular cells using ImageJ software.

**RNA isolation and Real-time PCR**

Total RNA samples were extracted using TRIzol reagent (Thermo Fisher, Waltham, MA, USA). The amount and purity of the RNA were determined on a NanoDrop 2000C Spectrophotometer (Thermo Fisher, Waltham, MA, USA). For real-time PCR, elongation factor-1 alpha (*ef1a*) was used as the internal standard for target genes. All of the primers used in this study are listed in Supplementary Table 1. Real-time PCR was carried out as previously described by (*2*).

**Expression and purification of recombinant IL-1β**

The zebrafish *il-1β* cDNA fragment corresponding to the mature peptide was synthesized and cloned into the pET30a expression vector. The IL-1β recombinant protein was expressed and purified from *E.coli* bacterial system (Genscript, China).

**Antibody Production**

A peptide (CRQNLFDEKSVTRTG) of zebrafish Nlrp1 was selected as antigen for the production of polyclonal antibody (Genscript, China). The synthetic peptides were used to immunize New Zealand white rabbits for antibody production (Genscript, China).

**Western blot analysis**

30 preovulatory follicles at different time points after hCG injection (20 IU/fish) were collected and lysed, then follicle lysates were clarified by centrifugation at 12,000 rpm for 10 min at 4°C, the supernatant was transferred to a new tube, concentrations were assessed by BCA protein assay (Beyotime, Shanghai, China). After boiling of samples in 4×protein loading buffer plus dithiothreitol (DTT), 20μg protein were loaded and electrophoresed on 12% SDS-PAGE gels. The separated proteins were transferred onto polyvinylidene difluoride membranes (PVDF, 0.45μm, Millipore, Bedford, MA, USA) and blocked with 5% blocking agent (Becton, Dickinson and Company, Ranklin Lakes, NJ, USA), then immunoblotted with primary antibodies at 4°C overnight with rotation. The protein bands were visualized by a Western blotting kit (Millipore, Billerica, MA, USA) after incubation with secondary antibody conjugated with horseradish peroxidase. Horseradish peroxidase (HRP)-labeled secondary antibodies were used to generate a chemiluminescent signal which was detected with a CCD camera-based imager (Chemidoc MP Imaging System, Bio-Rad, Hercules, CA, USA).

**Isolation of ovarian follicles and primary culture of ovarian follicular cells**

The staging system used is based on the original definition of Selman (*3*). Different stages of follicles from ovaries were dissected in 60% Leibovitz L-15 medium as described by us (*4*). Primary culture of zebrafish ovarian follicular cells was performed according to our established protocol (*5*). Briefly, follicles of the vitellogenic stage from 15 to 25 females were carefully selected and cultured in 25 cm^2^ flask for 6 days in M199 medium plus 10% fetal bovine serum (FBS) under the conditions of 28°C and 5% CO_2_. The cells were sub-cultured in 24-well plates at a density of 100,000 cells per well for 24 hours before hormone and drug treatment.

**Ovarian follicle incubations**

Follicles of full grown but immature (FG; about 0.65 mm in diameter) were manually isolated from adult zebrafish ovary. Follicles were incubated (around 30 follicles/well) in 24-well culture plates at 28°C. After treatment, follicles that underwent GVBD were identified by their ooplasmic clearing (due to proteolytic cleavage of vitellogenin). Each group had five replicate wells and each experiment was repeated at least three times.

**Intraperitoneal injection into adult zebrafish**

The procedure of Kinkel *et al*. (*6*) was followed with minor modifications. Briefly, after fasting and anesthetization, zebrafish were quickly placed on an agar gel plate. Using a microinjection system (WPI, USA), 2 µl hCG (10 IU/µl) with or without IL-1β (3 ng or 125 ng/fish), VX-765 (20 or 100 µM, 4 µl/fish), Ac-FEID-CMK (10 or 25 mM, 4 µl/fish), UCC950 (100mM, 4 µl/fish) was carefully injected into the midline between the pelvic fins. After injection, the fish were immediately transferred back to the aquarium for recovery. At least ten fishes per group was used to determine the effects of proteins and drugs on ovulation rate.

**Transmission electron microscopy and morphometry**

Transmission electron microscopy (TEM) was performed at the Electron Microscopy Center of Lanzhou University (Lanzhou, China). For TEM sample preparation and imaging, We collected 1mm^3^ follicles from the adult zebrafish ovary after hCG injection (20 IU/fish, 0 h, 1.5 h, 2 h, 2.5 h) and immediately fixed it in 2.5% glutaraldehyde (0.2 M phosphate buffer saline 25 ml pH 7.4, the aqueous solution of 25% glutaraldehyde 5 ml, add double distilled water to 50 ml) overnight at 4℃. Next day discard the fixative solution, wash the samples 5 times with PBS (about 1 hour). Then postfixed in 1% osmium tetroxide for 1h at 28℃. Follicles were dehydrated with graded ethanol solutions followed by acetone (30%-70%-90%-100% ethanol for 6min; 90%, 100% acetone 3 times), then cleaning by propylene oxide (Resin:Epon=1:3; Resin:Epon=3:1 respectively for 15min), embedded in an Epon812-resin mixture (Epon812 16.2 g, DDSA 10.0 g, MNA 8.9 g, DMP-30 0.35 g) for 30min, and there after solided more than 48h at 60℃. Silver sections were cut with a glass knife (Leica EM KMR3, Germany), Semithin sections (1 μm) were cut and observed to ensure structural integrity before thin sections (60 nm) performed on an MT-X ultratome (Leica EM UC7, Germany). The samples were stained with uranyl acetate and lead citrate for 40min (Leica EM AC20, Germany). and examined with a transmission electron microscope (FEI T12, USA) at 120 kV.

**Targeted gene disruption of *gsdmeb* by CRISPR/Cas9 system**

sgRNA (seed sequence is GGACTGCAGTTTGCTGGATC) for zebrafish *gsdmeb* was synthesized from Genscript. Cas9 protein was purchased from Takara. The CRISPR-Cas9 system preparation and microinjection into one-cell stage zebrafish embryos were conducted according to a protocol (*7*). The injected embryos were allowed to develop to adulthood followed by outcross with wild type fish. Ten F1 embryos generated from each pair of F0 fish were sacrificed for genomic DNA extraction for genotyping. F1 zebrafish embryos were then raised to adulthood, screened for heterozygotes, and then self-crossed to generate the F2 generation. Homozygous *gsdmeb* mutants were chosen by genomic PCR and confirmed by sequencing as described (*1*). Primers for *gsdmeb* screening were listed in Supplemental Table 1. For assessing the effects on ovulation, the ovulation rate was compared among the wild-type, heterozygous and homozygous mutants with the same developmental stage (3 months), body length and weight (four fishes were used for each genotype). The ovulation percentage in each fish was calculated as the number of ovulated oocytes/(number of ovulated oocytes + number of matured oocytes + number of FG stage follicles) as described (*8*).

**Statistical analysis**

All data are expressed as means ± SEM. P < 0.05 was considered statistically significant. We used one-way ANOVA, followed by Fisher’s least significant difference test. Statistical comparison of two groups was conducted using an unpaired two-tailed Students’*t* test. The statistical tests were in GraphPad InStat software (GraphPad Software, La Jolla, CA, USA).

**References**

1. L. Chu, J. Li, Y. Liu, W. Hu, C. H. K. Cheng, Targeted gene disruption in zebrafish reveals noncanonical functions of LH signaling in reproduction. *Mol Endocrinol* **28**, 1785-1795 (2014).

2. J. Li, Z. Liu, D. Wang, C. H. Cheng, Insulin-like growth factor 3 is involved in oocyte maturation in zebrafish. *Biol Reprod* **84**, 476-486 (2011).

3. K. Selman, R. A. Wallace, A. Sarka, X. Qi, Stages of oocyte development in the zebrafish, *Brachydanio rerio*. *Journal of Morphology* **218**, 22 (1993).

4. J. Li, W. Ge, Zebrafish as a model for studying ovarian development: Recent advances from targeted gene knockout studies. *Molecular and cellular endocrinology* **507**, 1-19 (2020).

5. J. Li, L. Chu, X. Sun, Y. Liu, C. H. Cheng, IGFs mediate the action of LH on oocyte maturation in zebrafish. *Mol Endocrinol* **29**, 373-383 (2015).

6. M. D. Kinkel, S. C. Eames, L. H. Philipson, V. E. Prince, Intraperitoneal injection into adult zebrafish. *J Vis Exp*, (2010).

7. C. E. Vejnar, M. A. Moreno-Mateos, D. Cifuentes, A. A. Bazzini, A. J. Giraldez, Optimized CRISPR-Cas9 system for genome editing in zebrafish. *Cold Spring Harb Protoc* **2016**, (2016).

8. J. Li, C. Niu, C. H. K. Cheng, Igf3 serves as a mediator of luteinizing hormone in zebrafish ovulation. *Biol Reprod* **99**, 1235-1243 (2018).

Fig. S1


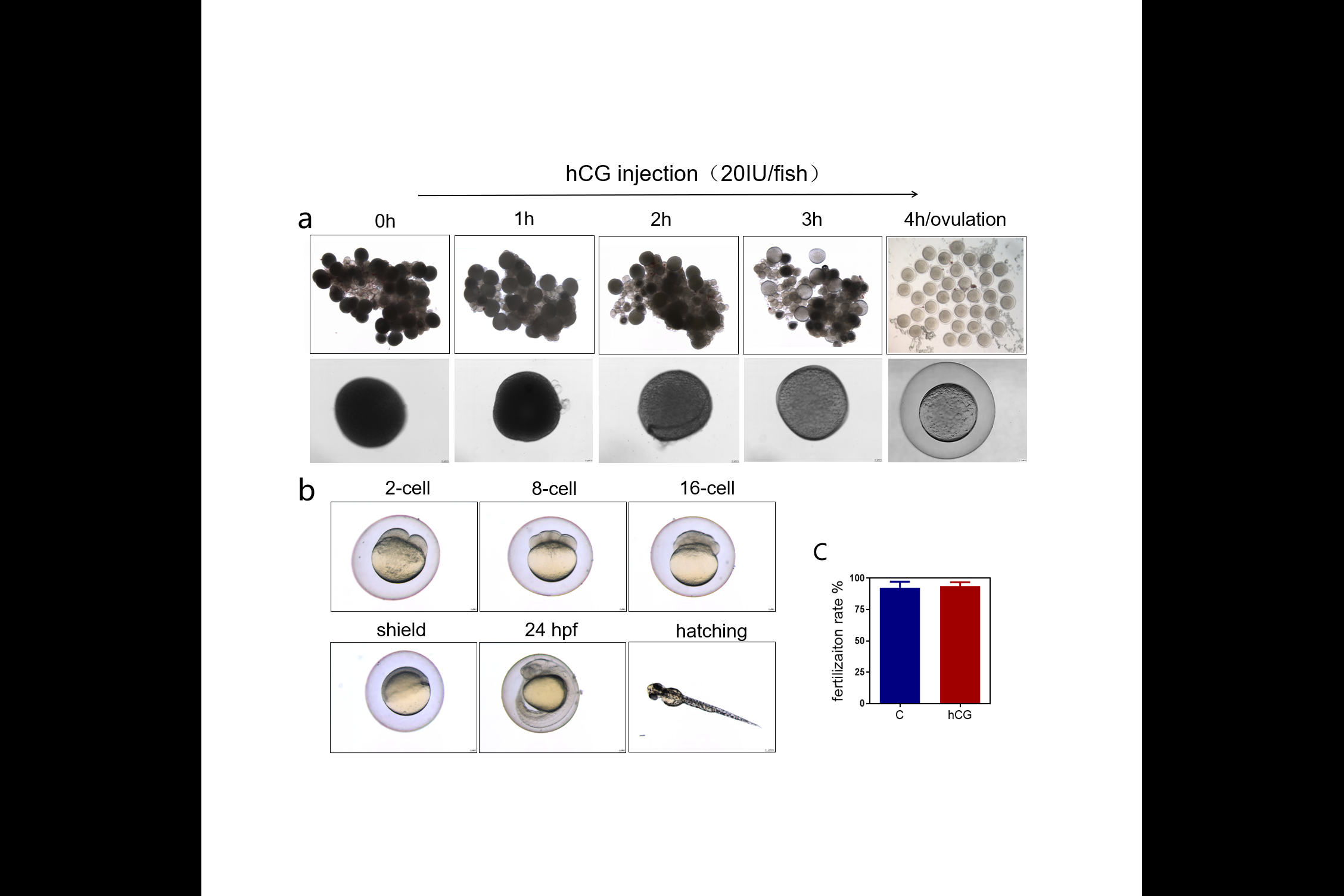


Fig. S1 Ovulation can be induced by administration of hCG. (a) A representative picture showing the morphology of the ovary and preovulatory/ovulatory follicles at different time points of the ovulation process artificially induced by administration of hCG (20 IU/fish). (b) A representative picture showing the normal early embryogenesis after fertilization of ovulatory oocyte obtained by administration of hCG (20 IU/fish). (c) Fertilization rate of ovulated oocyte induced by administration of hCG (20 IU/fish). Each value represents the mean ± SEM of triplicate assays from three independent experiments.

Fig. S2


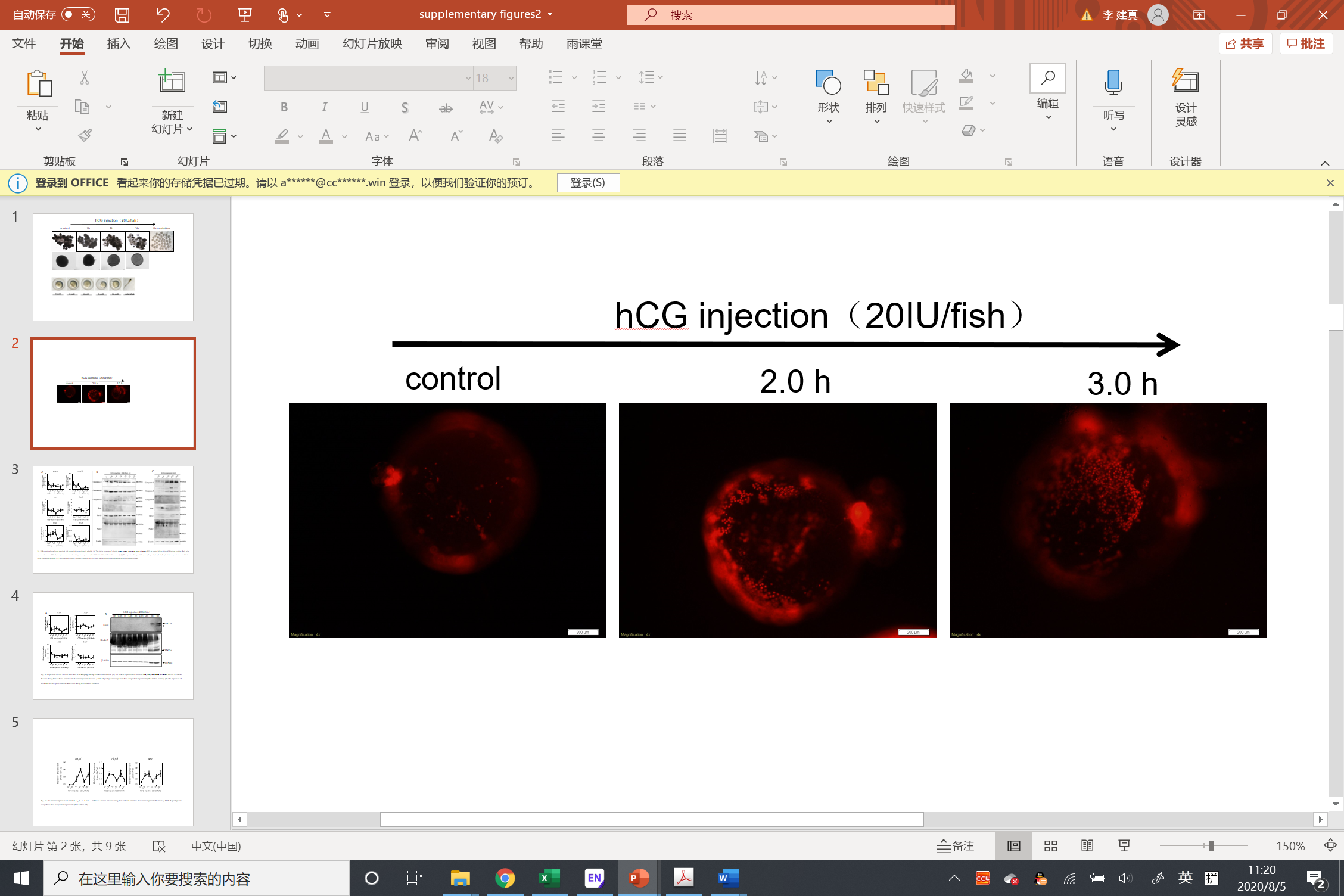


Fig. S2 PI staining of follicles at different time points of zebrafish ovulation after hCG injection (20 IU/fish). Images are representative of three independent experiments.

Fig. S3


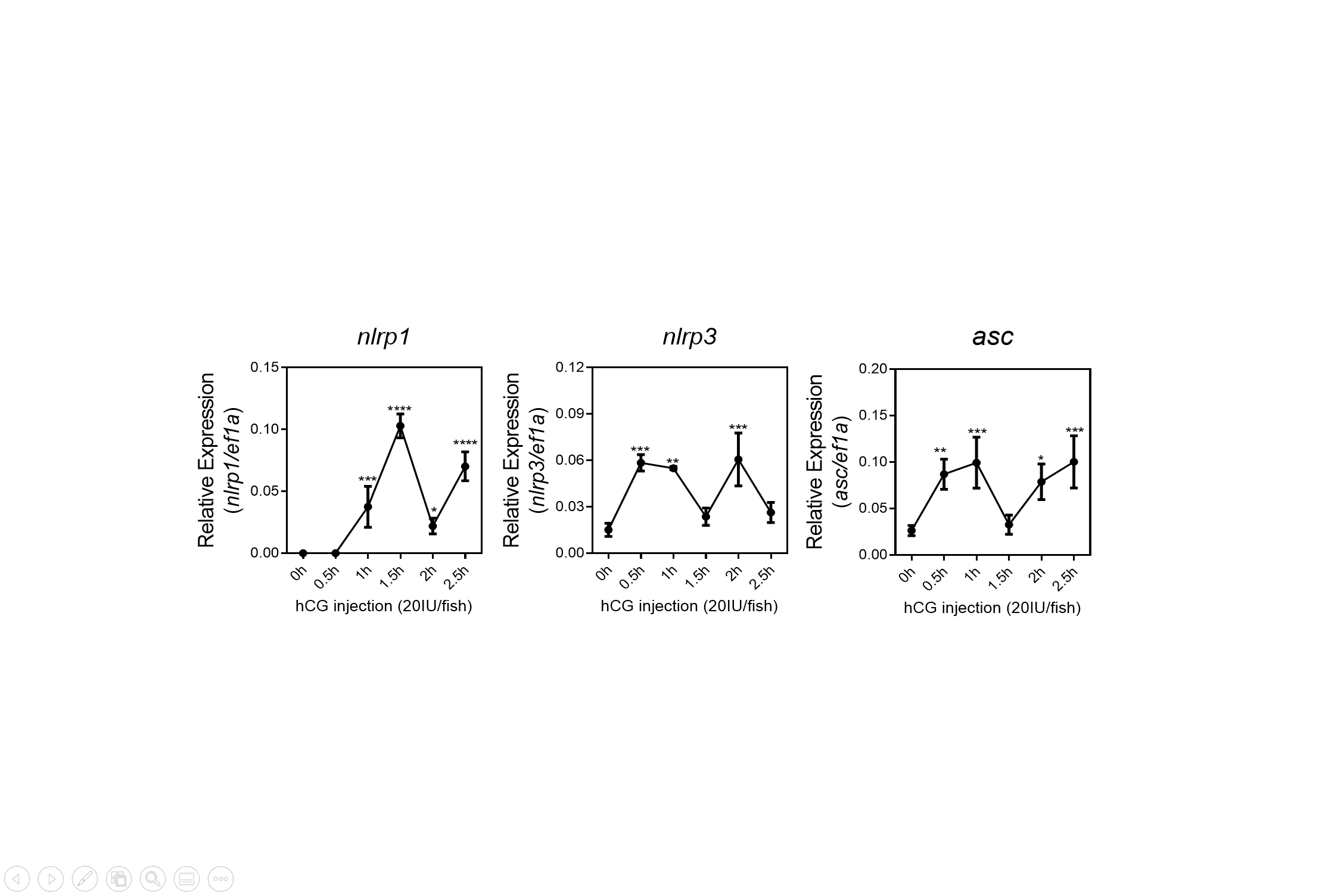


Fig. S3 The relative expression of zebrafish *nlrp1* and *nlrp3* mRNA in preovulatory follicles at different time points during hCG-induced ovulation. Each value represents the mean ± SEM of quintuplicate assays from three independent experiments (*P < 0.05, **P < 0.01, ***P < 0.001 and ***P < 0.0001 vs. 0 h).

Fig. S4


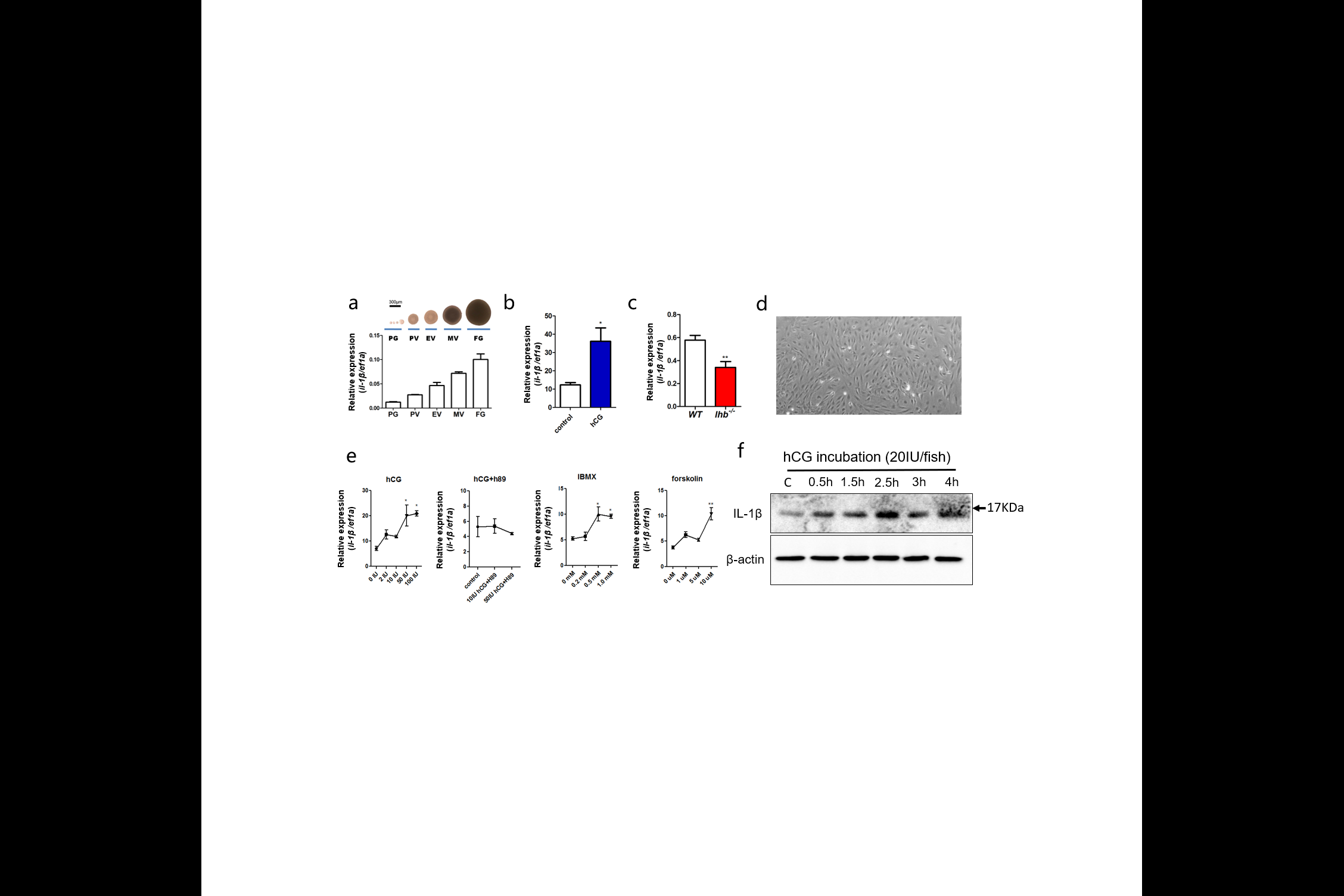


Fig. S4 The expression and regulation of IL-1β during ovulation of zebrafish. (a) The relative expression of *il-1β* mRNA during folliculogenesis of zebrafish. The above panel is the morphology of different stages of ovarian follicles during folliculogenesis of zebrafish. The bottom panel is the expression of *il-1β* mRNA in ovarian follicles from the PG to FG stage. PG, primary growth stage; PV, pre-vitellogenesis stage; EV, early vitellogenesis stage; MV, middle vitellogenesis stage; FG, fully grown stage. (b) The relative expression of *il-1β* mRNA in the follicular cell layer after administration of hCG (20 IU/fish) at 1 h. (c) The relative expression of *il-1β* mRNA in preovulatory follicles in wild type (WT) and luteinizing hormone beta subunit mutant (*lhb^-/-^*) zebrafish. (d) The morphology of primary cultured zebrafish follicular cells. (e) The change of *il-1β* mRNA in primary cultured zebrafish follicular cells by treatment with different concentrations of hCG (from 0 to 100 IU/ml), by co-treatment with hCG and a PKA inhibitor H89, by treatment with different concentration of a cAMP agonist IBMX, or by forskolin. Each value represents the mean ± SEM of quintuplicate assays from three independent experiments (*P < 0.05, **P < 0.01 vs. control). (f) The change of mature IL-1β protein by treatment with hCG in primary cultured zebrafish follicular cells.

Fig. S5


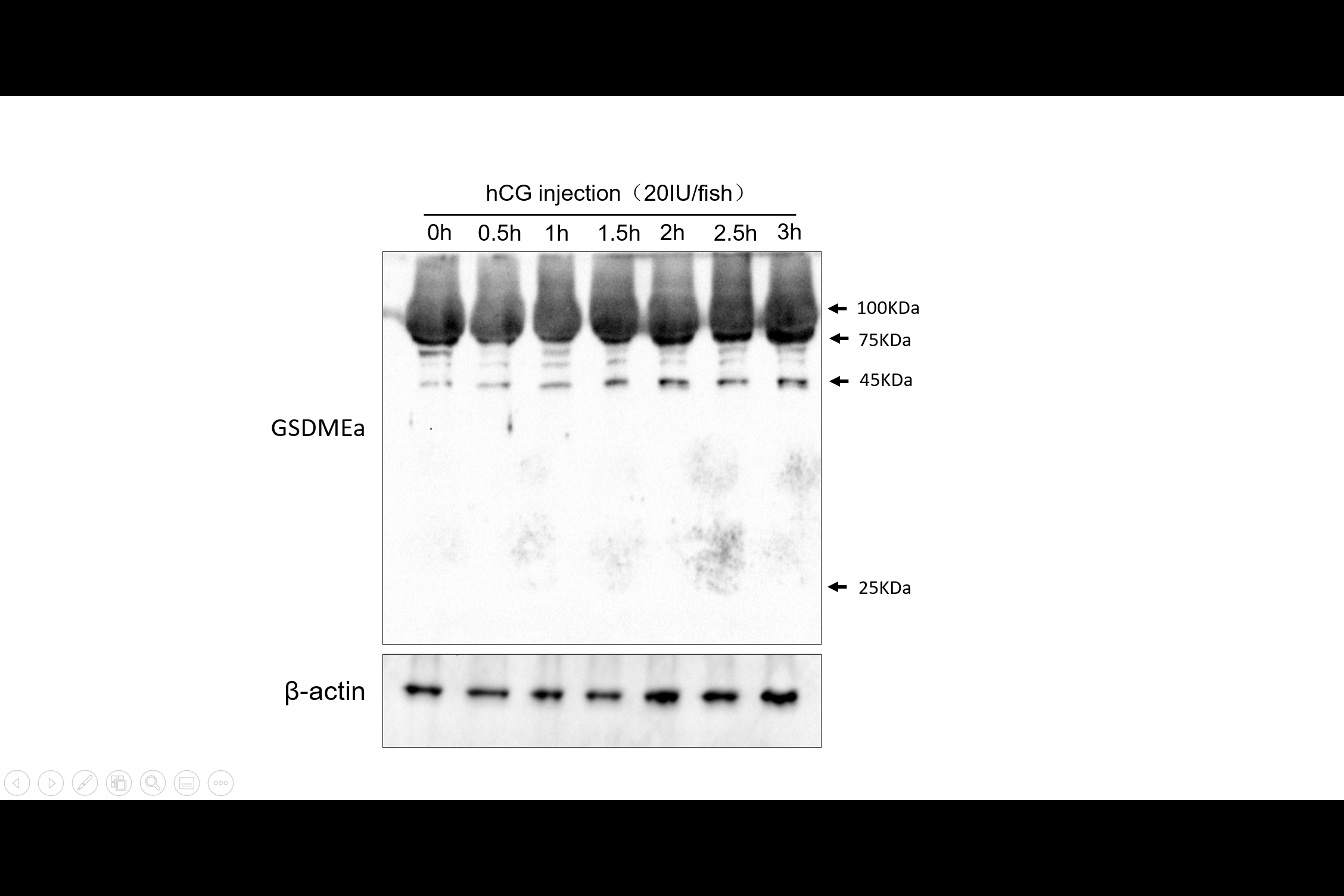


Fig. S5 The expression of zebrafish GSDMEa and β-actin protein in ovarian follicles during hCG-induced ovulation. Images are representative of three independent experiments.

Fig. S6


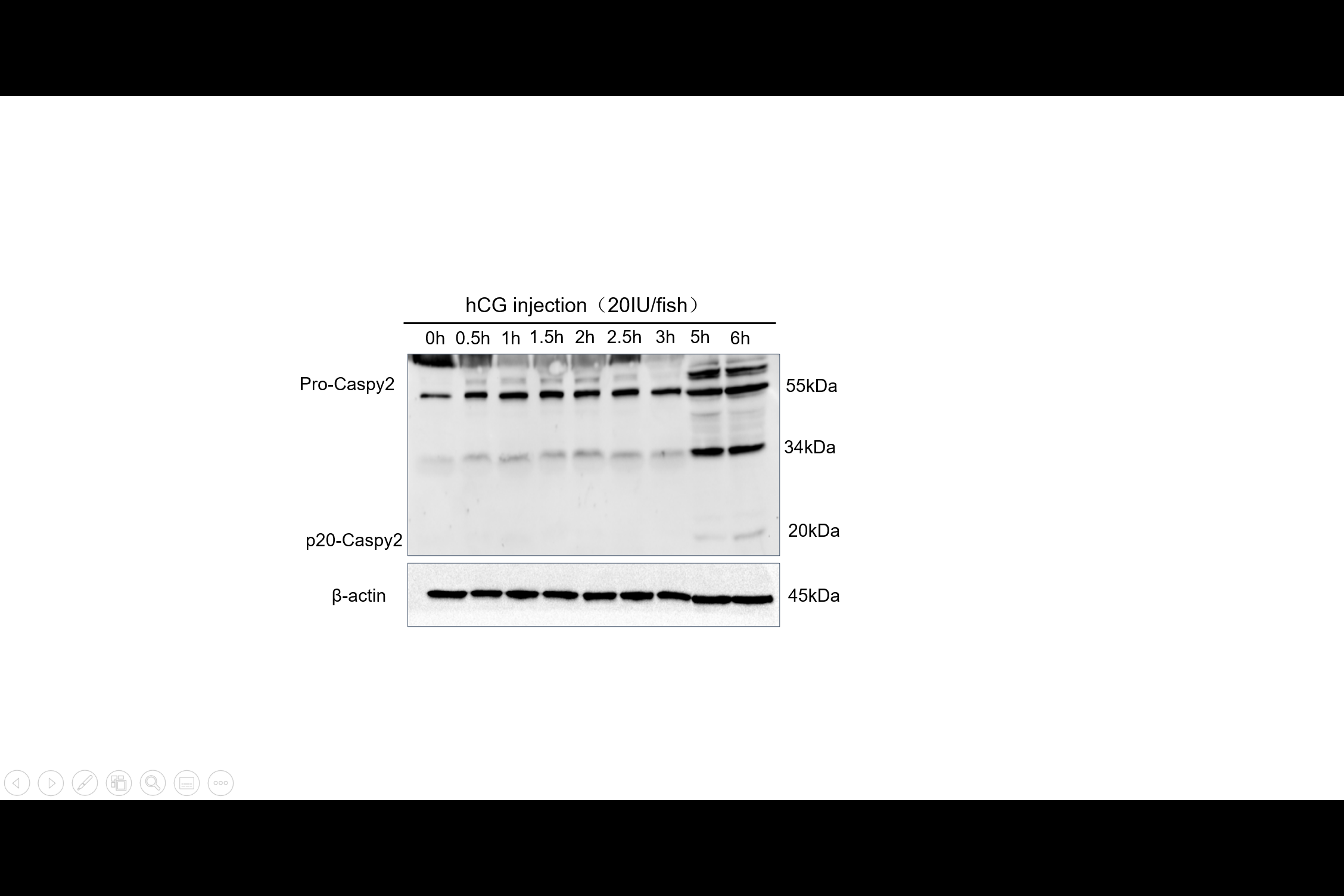


Fig. S6 The expression of zebrafish Caspy2 and β-actin protein in ovarian follicles during hCG-induced ovulation. Images are representative of three independent experiments.

Fig. S7


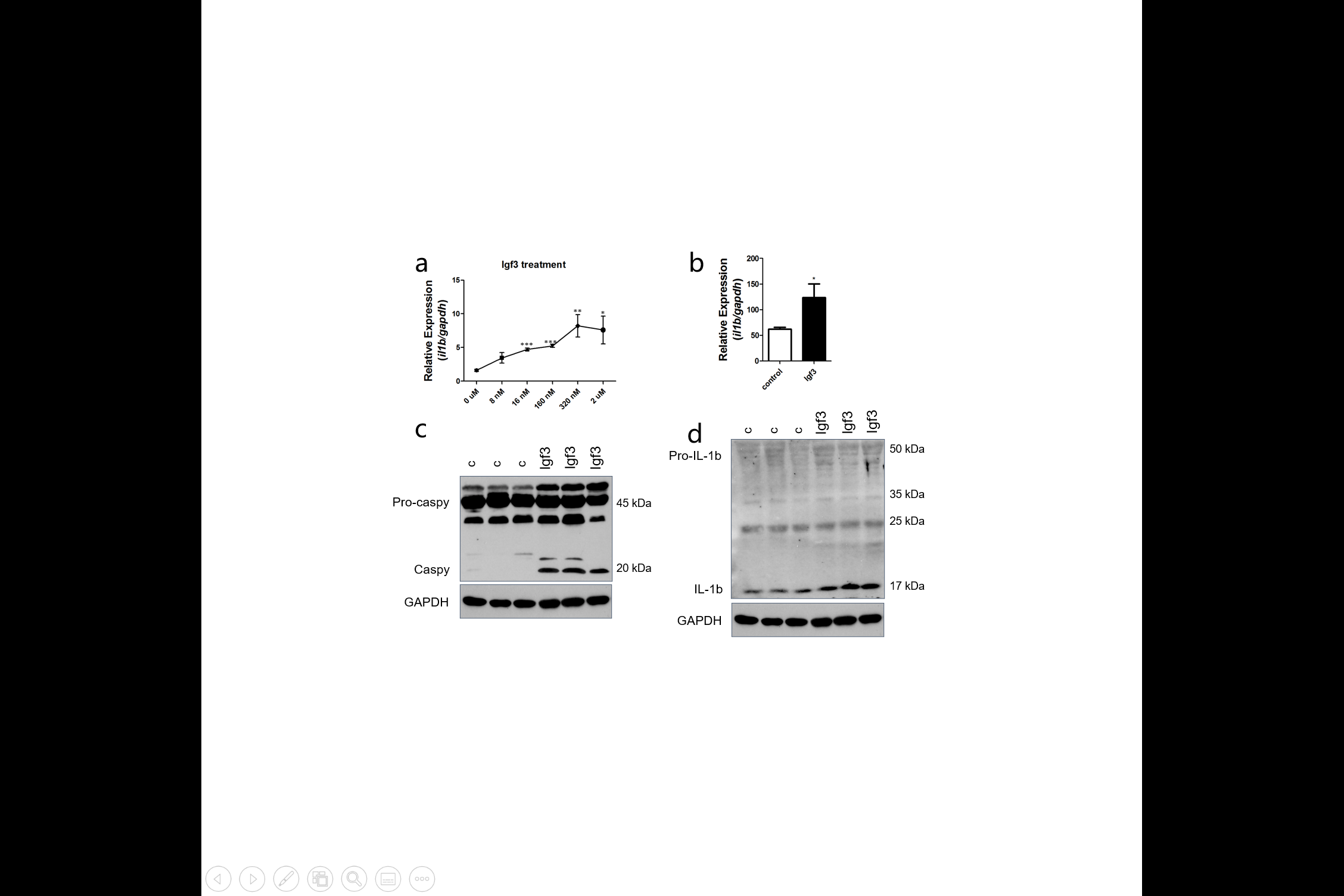


Fig. S7 Effects of Igf3 on expression of Caspase-1 and IL-1β in zebrafish follicles. (a) The relative expression of *il-1β* mRNA by treatment with different concentrations of zebrafish Igf3 recombinant protein (zIgf3) in primary cultured zebrafish follicular cells. Each value represents the mean ± SEM of quintuplicate assays from three independent experiments (*P < 0.05, **P < 0.01, ***P < 0.001 vs. 0 h or control). (b) The relative expression of *il-1β* mRNA in zebrafish follicular cell layer at 2 h by administration of zIgf3 protein (2 μg/fish). (c) The expression of Caspase-1 protein at 2 h after administration of zIgf3 protein (2 μg/fish). (d) The expression of IL-1β protein at 2 h after administration of zIgf3 protein. Images are representative of three independent experiments (2 μg/fish).

Fig. S8


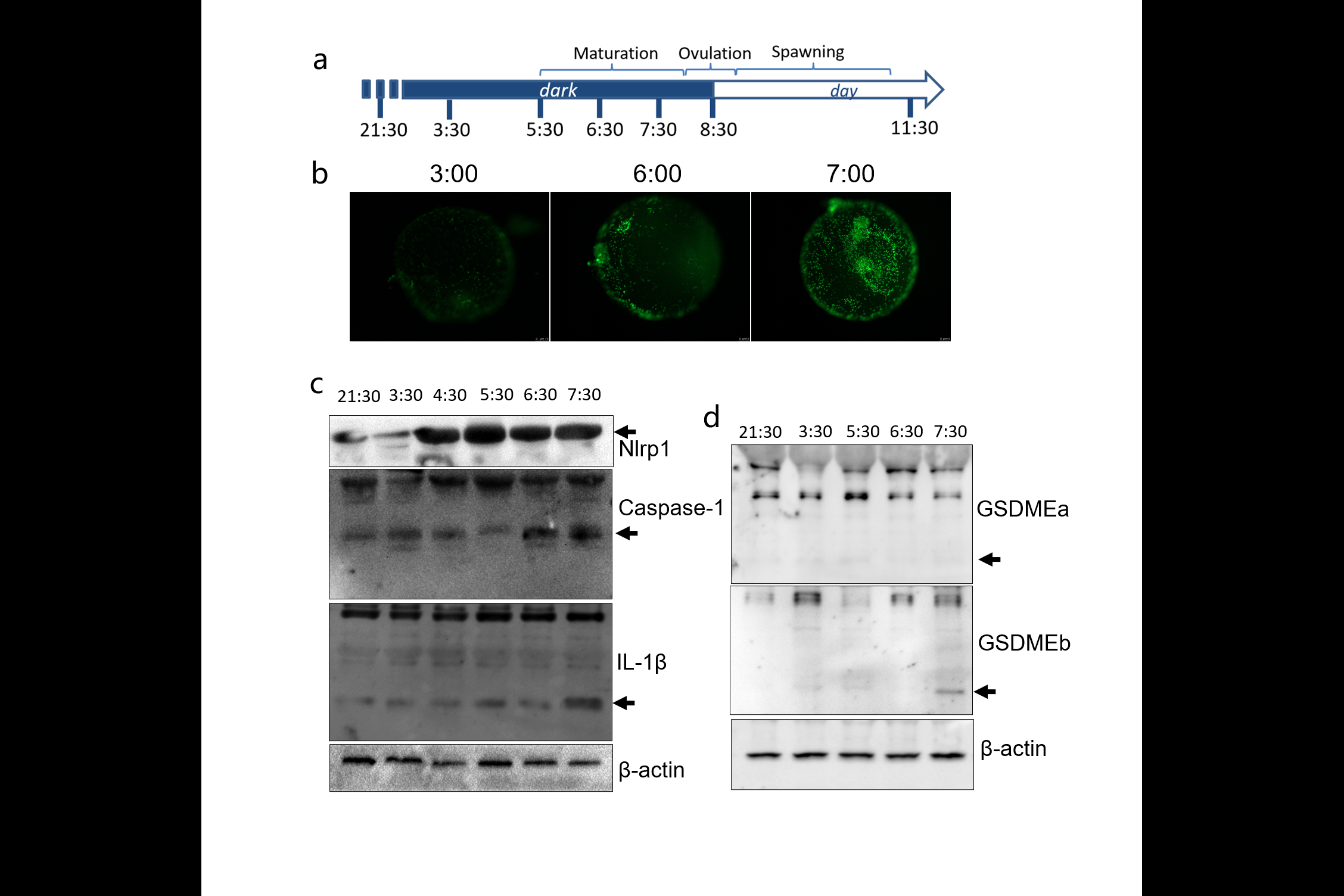


Fig. S8 The cell death and expression of pyroptosis-related genes in ovarian follicles during natural ovulatory cycle. (a) Schematic drawing showing the daily ovulatory cycle of zebrafish. Day and night cycles are indicated by open or black bars, respectively. Lights were switched on at 08:30 and off at 23:30. Approximate time for daily oocyte maturation, ovulation, and spawning in zebrafish are indicated on top of the bar. Samples were collected from six representative time points; (b) SYOTX Green staining of follicles at different time points of natural ovulatory cycle. Images are representative of three independent experiments; (c) The expression of Nlrp1, Caspase-1, IL-1β and β-actin protein in ovarian follicles at different time points during natural ovulatory cycle of zebrafish; (d) The expression of GSDMEa, GSDMEb and β-actin protein in ovarian follicles at different time points during natural ovulatory cycle of zebrafish.

Fig. S9


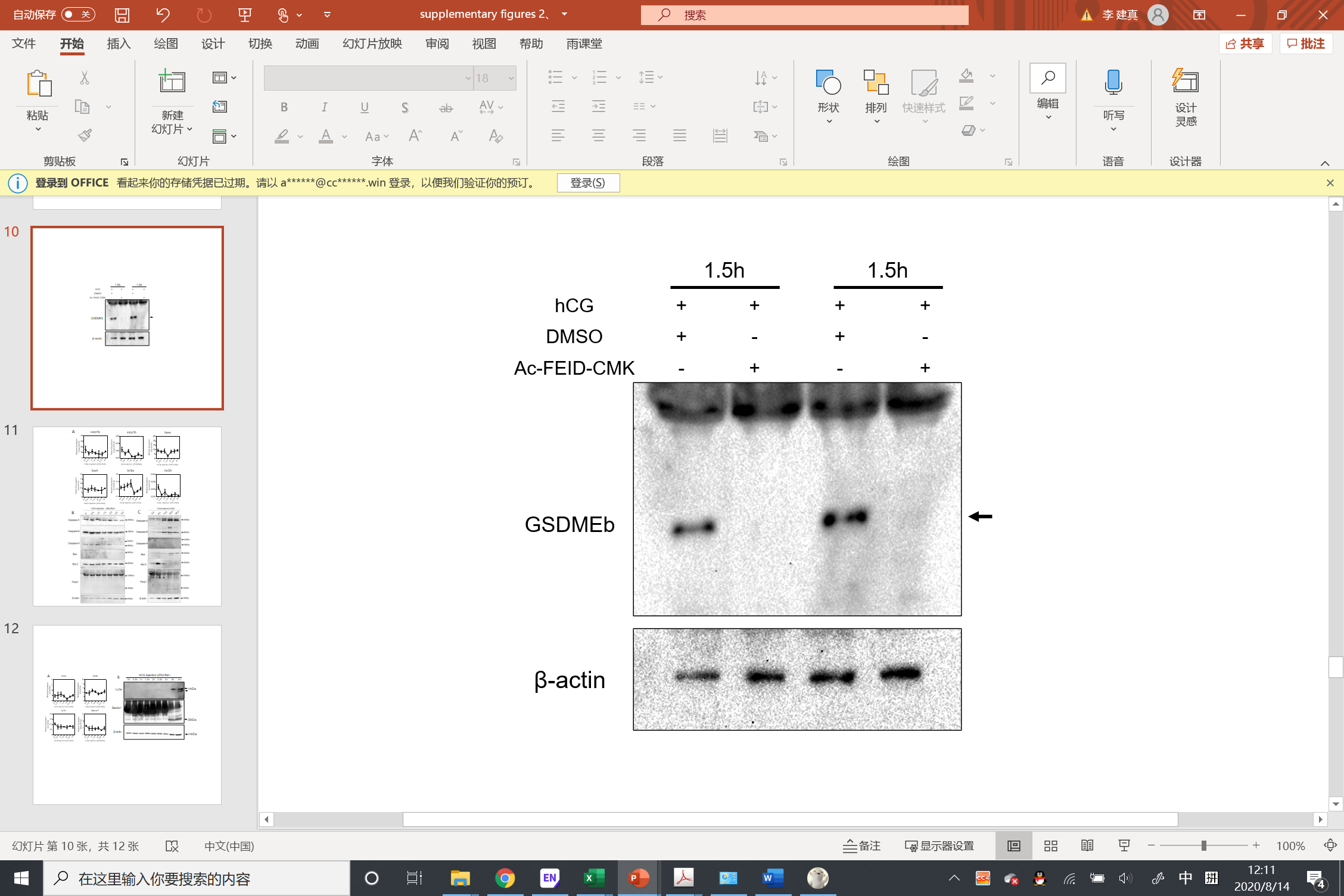


Fig. S9 The cleavage of zebrafish GSDMEb protein in ovarian follicles at 1.5 h after administration of hCG (20 IU/fish) with or without Ac-FEID-CMK (10 mM, 4 μl/fish).

Fig. S10


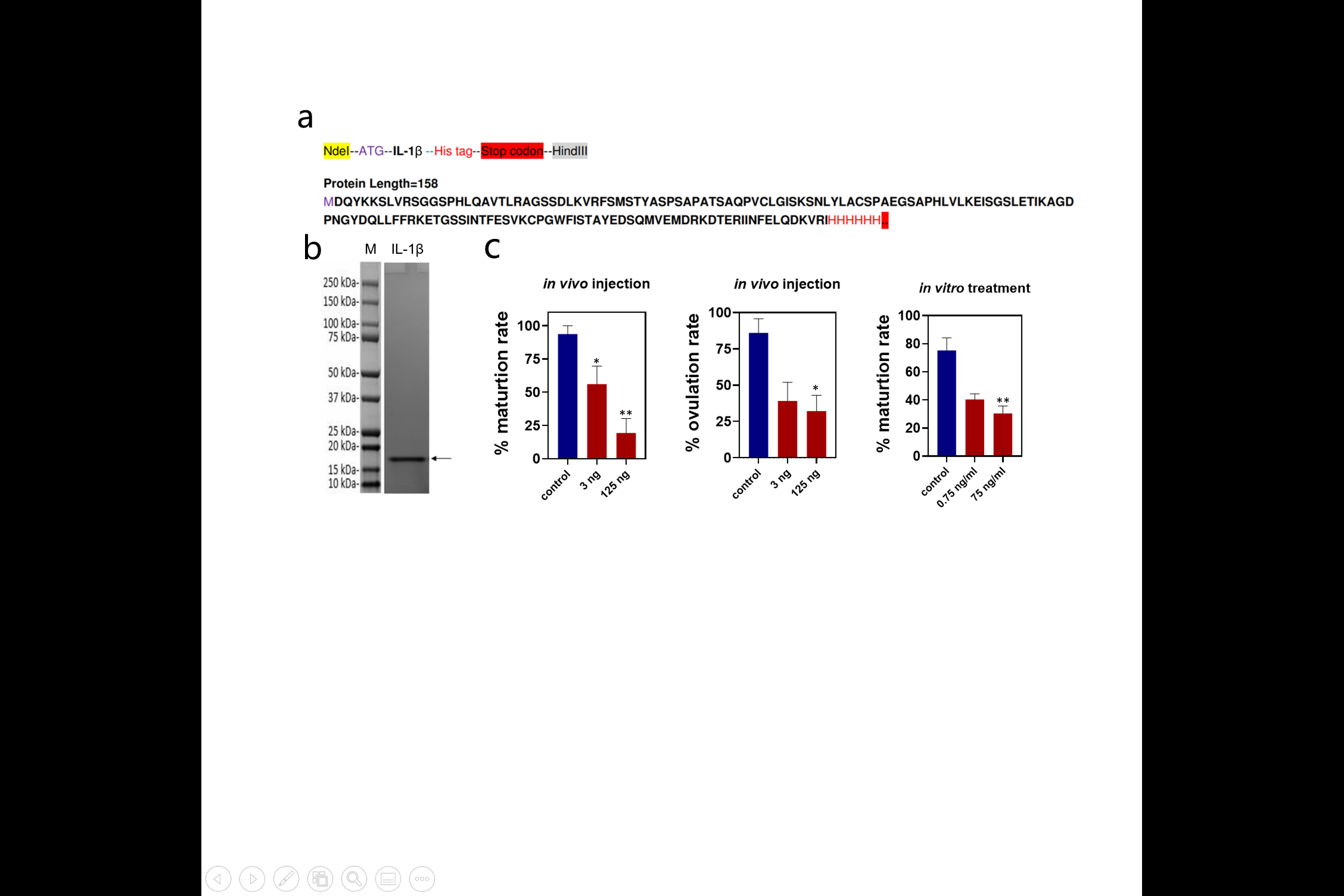


Fig. S10 The expression of zebrafish IL-1β recombinant protein and its effects on ovulation in zebrafish. (a) Schematic drawing showing the sequence of IL-1β mature protein and strategy for cloning; (b) The SDS-PAGE result of purified zebrafish IL-1β recombinant protein; (c) The effect of zebrafish IL-1β recombinant protein on hCG-induced oocyte maturation and ovulation. For *in vivo* injection, the maturation rate and ovulation rate were calculated by administration of hCG (10 IU/fish) with or without IL-1β recombinant protein (3 ng/fish or 125 ng/fish) for 5 hours. For *in vitro* treatment, the oocyte maturation ratio was calculated after treatment with IL-1β recombinant protein (0.75 ng/ml or 75 ng/ml) for 16 hours.

Fig. S11


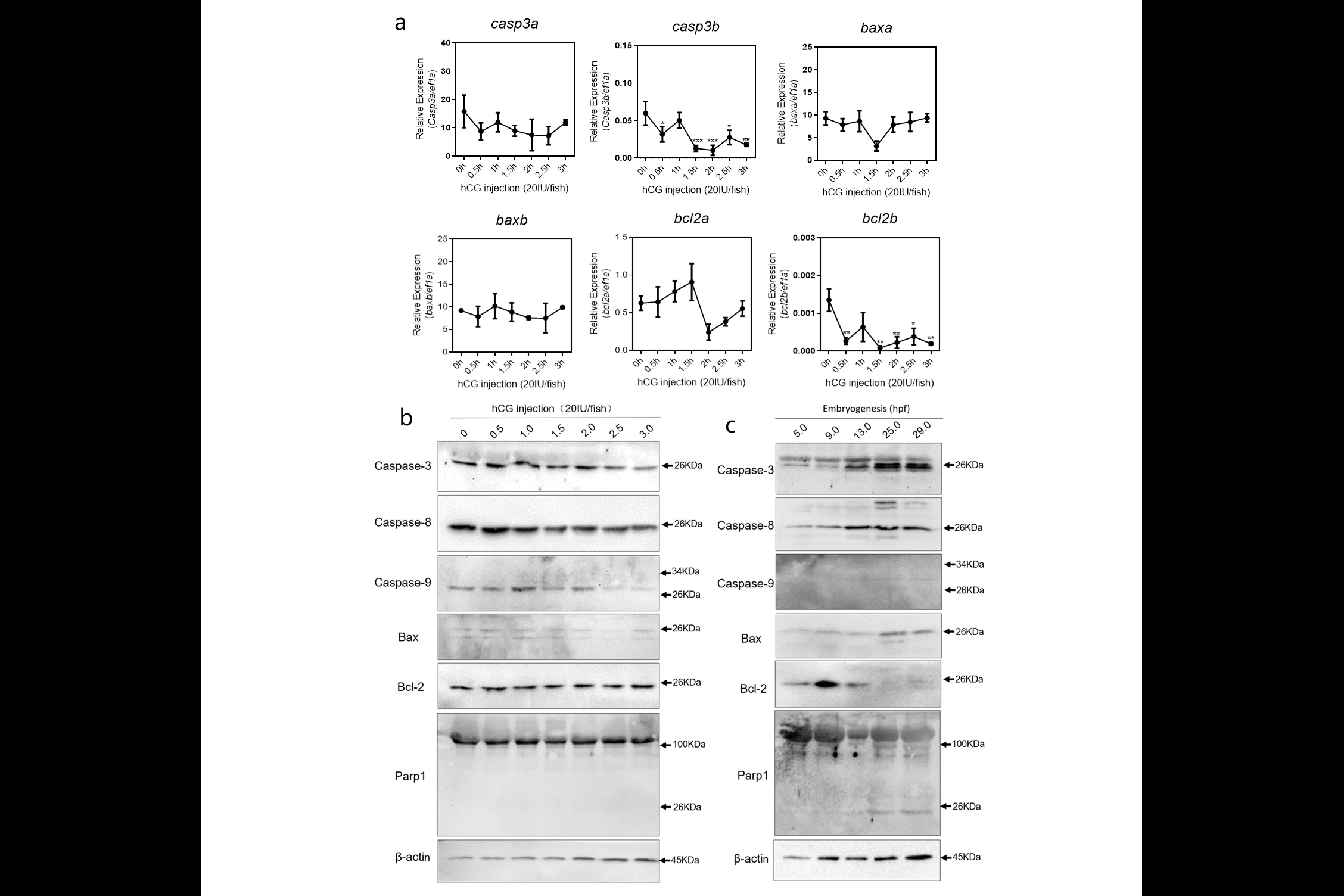


Fig. S11 Expression of core factors associated with apoptosis during ovulation in zebrafish: (A) The relative expression of zebrafish *caspase-3a* (*casp3a*), *caspase-3b* (*casp3b*), *baxa*, *baxb*, *bcl2a* and *bcl2b* mRNA in ovarian follicles during hCG-induced ovulation. Each value represents the mean ± SEM of quintuplicate assays from three independent experiments (*P < 0.05, **P < 0.01, ***P < 0.001 vs. 0 h). (B) The expression of Caspase-3, Caspase-8, Caspase-9, Bax, Bcl-2, Parp1 and β-actin protein in ovarian follicles during hCG-induced ovulation. (C) The expression of Caspase-3, Caspase-8, Caspase-9, Bax, Bcl-2, Parp1 and β-actin protein in ovarian follicles during hCG-induced ovulation.

Fig. S12


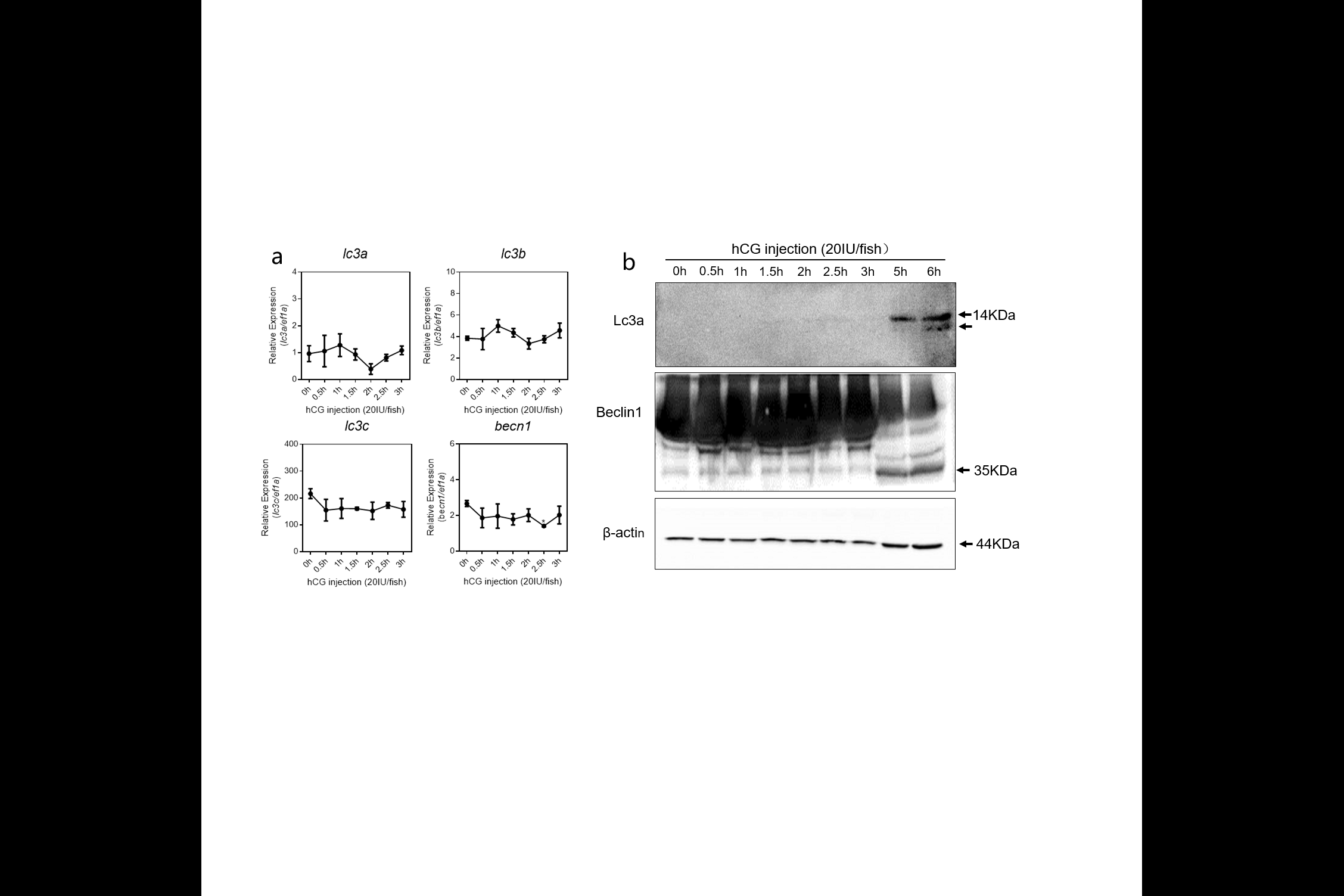


Fig. S12 Expression of core l factors associated with autophagy during ovulation in zebrafish: (a) The relative expression of zebrafish *lc3a*, *lc3b*, *lc3c* and *becn1* mRNA in ovarian follicles during hCG-induced ovulation. Each value represents the mean ± SEM of quintuplicate assays from three independent experiments (*P < 0.05 vs. control). (b) The expression of Lc3a and Beclin 1 protein in ovarian follicles during hCG-induced ovulation.

**Supplementary Table 1**. Primers used in this study.

| Gene | Sequence (sense) | Sequence (antisense) | Application |
| --- | --- | --- | --- |
| *ef1a* | CTGGAGGCCAGCTCAAACAT | ATCAAGAAGAGTAGTACCGCTAGCATTAC | Real-time PCR |
| *il-1b* | CTGAAATGATGGCATGCGGG | TGTAGCTCATTGCAAGCGGA | Real-time PCR |
| *nlrp1* | GAGGAGATGAGCCTGACTGAGCTCTTG | TAATTCAGCAGCAGCGGTATATCTAGT | Real-time PCR |
| *nlrp3* | TGAACAGGTTGATGACTGATATGCT | ACAGCGATTTTCCCAGCATCCTTGC | Real-time PCR |
| *casp3a* | CCTGGTGTGGAAACTGACCA | TGAATGAACCATGAGCCGGT | Real-time PCR |
| *casp3b* | GTGACGGTGTAGGTGACGAG | GTCGCACAGCGAGGAGATAA | Real-time PCR |
| *baxa* | ACTGGATCAGGGAACAGGGT | TGCGAATCACCAATGCTGTG | Real-time PCR |
| *baxb* | ATCGGTGACAAACTCGACCA | AGCACGATAATTCTGCCCCA | Real-time PCR |
| *bcl-2a* | CGAGTTTGGTGGGACCATGT | CGTACATCTCCACGAAGGCA | Real-time PCR |
| *bcl-2b* | CGGGGCAGGTGGATAACATT | GCAGCTAGACCAAAGACCGT | Real-time PCR |
| *becn1* | AACAAACAAGATGGCGTGGC | GTCTGCGACTCAAGTTCTCCA | Real-time PCR |
| *gsdmeb* | CATTTTTGTCCACAGTTGTGGTTG | TCATGCCCTCATGCTCATGCT | Genomic PCR |
